# Prevalence and Clinical Profile of Severe Acute Respiratory Syndrome Coronavirus 2 Infection among Farmworkers, California, USA, June–November 2020

**DOI:** 10.3201/eid2705.204949

**Published:** 2021-05

**Authors:** Joseph A. Lewnard, Ana M. Mora, Oguchi Nkwocha, Katherine Kogut, Stephen A. Rauch, Norma Morga, Samantha Hernandez, Marcus P. Wong, Karen Huen, Kristin Andrejko, Nicholas P. Jewell, Kimberly L. Parra, Nina Holland, Eva Harris, Maximiliano Cuevas, Brenda Eskenazi, on behalf of the CHAMACOS-Project-19 Study Team

**Affiliations:** University of California, Berkeley, Berkeley, California, USA (J.A. Lewnard, A.M. Mora, K. Kogut, S.A. Rauch, S. Hernandez, M.P. Wong, K. Huen, K. Andrejko, N.P. Jewell, N. Holland, E. Harris, B. Eskenazi);; Universidad Nacional, Heredia, Costa Rica (A.M. Mora);; Clínica de Salud del Valle de Salinas, Salinas, California, USA (O. Nkwocha, N. Morga, M. Cuevas);; London School of Hygiene and Tropical Medicine, London, UK (N.P. Jewell);; University of Arizona, Tucson, Arizona, USA (K.L. Parra)

**Keywords:** respiratory infections, severe acute respiratory syndrome coronavirus 2, SARS-CoV-2, SARS, COVID-19, coronavirus disease, zoonoses, viruses, coronavirus, farmworkers, infection prevalence, serosurvey, essential workers, Salinas Valley, California, United States

## Abstract

During the ongoing coronavirus disease (COVID-19) pandemic, farmworkers in the United States are considered essential personnel and continue in-person work. We conducted prospective surveillance for severe acute respiratory syndrome coronavirus 2 (SARS-CoV-2) infection and antibody prevalence among farmworkers in Salinas Valley, California, during June 15–November 30, 2020. We observed 22.1% (1,514/6,864) positivity for SARS-CoV-2 infection among farmworkers compared with 17.2% (1,255/7,305) among other adults from the same communities (risk ratio 1.29, 95% CI 1.20–1.37). In a nested study enrolling 1,115 farmworkers, prevalence of current infection was 27.7% among farmworkers reporting >1 COVID-19 symptom and 7.2% among farmworkers without symptoms (adjusted odds ratio 4.16, 95% CI 2.85–6.06). Prevalence of SARS-CoV-2 antibodies increased from 10.5% (95% CI 6.0%–18.4%) during July 16–August 31 to 21.2% (95% CI 16.6%–27.4%) during November 1–30. High SARS-CoV-2 infection prevalence among farmworkers underscores the need for vaccination and other preventive interventions.

In response to the ongoing coronavirus disease (COVID-19) pandemic, the United States and other countries have implemented broad interventions to mitigate community transmission of severe acute respiratory syndrome coronavirus 2 (SARS-CoV-2) (*1*). Workers in food supply and other industries deemed essential to continuity of public health and safety have continued in-person work (*2*). COVID-19 outbreaks have been reported among various essential workforce groups, including employees in food processing facilities (*3*,*4*), but studies prospectively assessing risk for infection among essential workers involved in food production are lacking.

Agriculture and related food production industries comprise one of the lowest-paid sectors of the US economy; 29% of full-time workers earn an annual individual income of <$12,760 or $26,200 for a family of 4 (*5*). Agriculture in particular draws on a predominantly Latino immigrant workforce (*6*), who work longer hours, receive lower wages, and experience higher levels of household poverty than their US-born counterparts (*7*). Among immigrant farmworkers, ≈54% are undocumented and thus have reduced access to federal benefits under the Coronavirus Aid, Relief, and Economic Security Act (*8*). Working conditions, poverty, and immigration status have compounded legal and economic challenges faced by farmworkers during the COVID-19 pandemic (*9*,*10*).

We initiated surveillance of SARS-CoV-2 infection among farmworkers in Salinas Valley, California, to monitor the COVID-19 epidemic. We previously described impacts of the pandemic on economic well-being, mental health, and food insecurity within this population (A.M. Mora, unpub. data, https://doi.org/10.1101/2020.12.18.20248518). Here, we report on the prevalence of SARS-CoV-2 infection among farmworkers tested during June–November 2020 and on symptoms and antibody responses within a subset of farmworkers enrolled in a cross-sectional study.

## Methods

### Study Setting

The Salinas Valley is a 90-mile stretch of agricultural land in Monterey County, California; prominent farmed crops include leafy greens, berries, broccoli, artichokes, and wine grapes. The agricultural workforce comprises ≈50,000 resident farmworkers, and an additional ≈40,000 seasonal workers support the peak summer and fall seasons (*11*). The overall population of Salinas Valley is 75% Latino, and 30%–60% of the region’s farmworkers are believed to be undocumented (*12*). Severe overcrowding and household disrepair are common among farmworkers (*13*), and many live in multigenerational households (*14*) or in labor camps, vehicles, and informal dwellings (*15*). Many farmworkers travel long distances to work, often in shared trucks or buses, and might work in close proximity to one another. The living and working conditions of farmworkers have led to concern about the difficulty of preventing SARS-CoV-2 transmission among farmworkers and in their communities (*16*).

We undertook this study in partnership with Clínica de Salud del Valle de Salinas (CSVS), a federally qualified community and migrant health center in Monterey County. As the main healthcare provider for the region’s farmworkers and their families, CSVS operates a network of 12 comprehensive primary care centers serving >52,000 low-income, primarily Spanish-speaking patients. The study was reviewed and approved by the Committee for Protection of Human Subjects at University of California, Berkeley.

### SARS-CoV-2 Testing

Testing for SARS-CoV-2 infection at CSVS clinics began on June 15, 2020, and was offered to all persons at clinics during weekday business hours. Medical personnel collected oropharyngeal specimens for detection of SARS-CoV-2 RNA via the qualitative Aptima SARS-CoV-2 Assay (Hologic, https://www.hologic.com), a nucleic acid transcription-mediated amplification (TMA) assay with an analytical sensitivity of 62.5 RNA transcript copies/mL (*17*) and clinical specificity of 99.9% (*18*). Patients receiving care from CSVS for any reason were encouraged by their healthcare providers to receive SARS-CoV-2 testing, regardless of symptoms; testing also was made available to persons who were not CSVS patients. No-cost testing for persons without insurance was supported by funding from the US Department of Health and Human Services Health Resources and Services Administration. In addition, CSVS conducted outreach testing via mobile testing facilities at community sites including low-income and employer-provided housing, agricultural fields, homeless shelters, food banks, and CSVS-run health fairs where free SARS-CoV-2 testing was offered alongside seasonal influenza vaccination and food donations.

### Clinical Surveillance Study

As part of routine clinical intake, all patients >18 years of age were asked about employment. We considered farmworkers to include all persons engaged in work in agriculture, including crop, nursery, and greenhouse laborers; agricultural equipment operators; workers in packing sheds and other food processing facilities; and farm and ranch animal workers and breeders.

### Cross-Sectional Study

#### Enrollment 

To determine the distribution, dynamics, and clinical profile of infection among farmworkers, we invited farmworkers who were receiving a SARS-CoV-2 TMA test at CSVS to participate in a more in-depth cross-sectional study during July 16–November 30, 2020. This study included SARS-CoV-2 antibody testing and a detailed questionnaire. To advertise the study, Spanish- and English-language fliers were designed describing the opportunity to receive free SARS-CoV-2 testing from CSVS and participate in the study. The fliers were hung in CSVS clinics and distributed in the community and to area growers. We stationed the study team at CSVS testing facilities and aimed to approach all patients receiving SARS-CoV-2 TMA tests to screen for study eligibility and invite them to participate in the cross-sectional study. When time allowed, study personnel called patients who had scheduled SARS-CoV-2 testing appointments at CSVS on the day before their visit to advertise the study and screen for eligibility. Participants in an ongoing longitudinal study of farmworker families (*12*) and those living in housing for farmworkers also were invited to participate and to bring other farmworkers.

Eligible participants were nonpregnant adult farmworkers >18 years of age receiving SARS-CoV-2 TMA testing at CSVS. Participants were eligible if they had conducted farm work <14 days before their testing date, had not participated previously, and spoke sufficient English or Spanish to give consent and complete study procedures. To accommodate the end of the growing season, from October 5 onward we enrolled persons who had engaged in farm work any time since March 2020.

#### Study Procedures 

The study team obtained a blood sample from each participant by venipuncture, measured participants’ height by using large-print tape measurers adhered to a post or wall, and measured their weight by using digital scales. The study team administered a 45-minute computer-guided questionnaire by telephone in Spanish or English within 48 hours of the enrollment visit and before SARS-CoV-2 testing results were available. Questionnaire items addressed participant demographics, socioeconomic status, symptoms since December 2019 and in the 2 weeks preceding enrollment, COVID-19 risk factors and exposures, and impacts of the pandemic on daily life and wellbeing (A.M. Mora et al., unpub. data, https://doi.org/10.1101/2020.12.18.20248518; A.M. Mora et al., unpub. data, https://doi.org/10.1101/2021.02.01.21250963). After participants completed all components of the study, the study team provided a $50 incentive via prepaid gift cards.

Blood specimens were stored immediately at 4°C–7°C and centrifuged <48 hours after collection. After centrifugation, plasma aliquots were heat-inactivated at 56°C for 30 minutes and stored at –80°C, then used for assessment of IgG reactivity against the SARS-CoV-2 spike protein via in-house ELISAs (*19*). In brief, recombinant full-length SARS-CoV-2 spike protein (courtesy of John Pak, Chan Zuckerberg Biohub, San Francisco, California) was coated on Nunc Maxisorp ELISA plates (Thermo Fisher Scientific, https://www.thermofisher.com) at 1.5 µg/mL. Plates were blocked with 2.5% nonfat dry milk in 1× phosphate-buffered saline (PBS) for 2 hours at 37°C. Plates were then washed 3 times in 1× PBS. Plasma samples diluted 1:100 in 1% nonfat dry milk in 1× PBS were added to the plate in duplicate wells. After a 1-hour incubation at 37°C, plates were washed 5 times in 1× PBS with 0.05% Tween-20 (Millipore Sigma, https://www.sigmaaldrich.com). Bound antispike IgG was detected by using horseradish peroxidase-conjugated goat anti-human IgG (Thermo Fisher Scientific). Plates were developed by using a 3,3′,5,5′-tetramethylbenzidine solution, and the reaction was stopped with 2 mol sulfuric acid after 6 minutes. We performed prior assay validation using convalescent serum samples collected >8 days post symptom onset from 60 hospitalized, PCR-confirmed COVID-19 cases, 57 of which were mild or subclinical and serum samples collected before 2020 from 131 unexposed persons. 

We considered specimens positive for anti-SARS-CoV-2 spike IgG if the ELISA optical density (OD) value was >0.096. This cutoff maximized area under the receiver operating characteristic curve, yielding 94.0% sensitivity and 98.5% specificity. We processed all specimens in duplicate; conducted reflex testing if >1 OD measurement fell in the borderline range of 0.07–0.3 or if the coefficient of variation between replicates was >30% and >1 OD measure was >0.07. We confirmed positive specimens by noting presence of IgG against the receptor-binding domain (RBD) of SARS-CoV-2 spike protein (courtesy of John Pak, Chan Zuckerberg Biohub) using the protocol described above and substituting the coating antigen with RBD at 3 µg/mL. We considered specimens positive when RBD ELISA OD values were >0.205, determined via a similar validation process as described above for spike protein.

### Statistical Analyses

#### Clinical Surveillance Study 

We tabulated results for all patients tested at CSVS during June 15–November 30, 2020, by age, sex, and farmworker status. We also computed 2-week moving averages in the daily proportion of tests yielding positive results and estimates of the final proportion of positive tests by patient age, sex, and farmworker status. We used beta distribution to define 2.5% and 97.5% quantiles for the proportion positive.

Cross-Sectional Study

We computed adjusted odds ratios (aORs) using logistic regression models accounting for age, sex, and venue to determine the association of symptoms experienced in the previous 2 weeks with a positive test result. We used the same logistic regression framework to estimate aORs for the association of each symptom experienced in the prior 2 weeks or at any time since December 2019 with continuous SARS-CoV-2 antibody OD measures.

We computed stabilized sampling weights (*20*) to correct for differences in the population enrolled in the study over time when estimating prevalence of infection to generate weights for each recruitment period, July 16–August 31, September 1–30, October 1–31, or November 1–30. We fit a multinomial logistic regression model that included a list of possible exposures (Table 1), the number of symptoms participants reported in the preceding 2 weeks, and the recruitment venue as predictors.

**Table 1:** Place of residence, living conditions, and working and transportation conditions that could lead to SARS-CoV-2 exposure among farmworkers enrolled in a cross-sectional study, Monterey County, California, USA, July 16–November 30, 2020*

Characteristics	Enrollees, no. (%)		
	All, n = 1,115	Clinic, n = 565	Outreach, n = 550
Community of residence			
Salinas	492 (44.1)	263 (46.5)	229 (41.6)
Northern Monterey County	73 (6.5)	18 (3.2)	55 (10.0)
Southern Monterey County	539 (48.3)	284 (50.3)	255 (46.4)
Outside Monterey County	11 (1.0)	0	11
Household size	n = 1,115	n = 565	n = 550
0 others	12 (1.1)	8 (1.4)	4 (0.7)
1–3 others	399 (35.8)	187 (33.1)	212 (38.6)
4–6 others	515 (46.2)	259 (45.8)	256 (46.5)
>7 others	189 (17.0)	111 (19.7)	78 (14.2)
Children in household	n = 1,114	n = 565	n = 549
Any children	836 (75.0)	440 (77.9)	396 (72.1)
Children attending school or daycare	n = 1,111	n = 562	n = 549
Any children	85 (7.7)	57 (10.1)	28 (5.1)
Residential overcrowding	n = 1,115	n = 565	n = 550
<2 persons/bedroom	490 (44.0)	224 (39.7)	266 (48.4)
>2 to <4 persons/bedroom	510 (45.7)	289 (51.2)	221 (40.2)
>4 persons/bedroom	115 (10.3)	52 (9.2)	63 (11.5)
Ability to isolate at home if infected	n = 1,115	n = 565	n = 550
Live alone or have >1 bedroom and bathroom	643 (57.7)	330 (58.4)	313 (56.9)
Size of company	n = 939	n = 574	n = 456
<25 workers	108 (11.5)	49 (10.1)	59 (12.9)
25–49 workers	132 (14.1)	67 (13.9)	65 (14.3)
50–499 workers	447 (47.6)	229 (47.4)	218 (47.8)
>500 workers	252 (26.8)	138 (28.9)	114 (25.0)
Work setting	n = 1,114	n = 564	n = 550
Indoors only	192 (17.2)	103 (18.3)	89 (16.2)
Outdoors only	849 (76.2)	425 (75.4)	424 (77.1)
Indoor and outdoor	73 (6.6)	36 (6.4)	37 (6.7)
Type of agricultural work	n = 1,105	n = 555	n = 550
Working in the fields	830 (74.4)	416 (73.6)	414 (75.3)
Packing shed	133 (11.9)	65 (11.5)	68 (12.4)
Processing facility	64 (5.74)	34 (6.0)	30 (5.5)
Nursery	40 (3.6)	18 (3.2)	22 (4.0)
Truck driver	38 (3.4)	19 (3.4)	19 (3.5)
Packing truck	22 (1.97)	15 (2.7)	7 (1.3)
Other	21 (1.88)	12 (2.1)	9 (1.6)
Commute to work	n = 1,088	n = 554	n = 534
Alone or with household members only	714 (65.6)	341 (61.6)	373 (69.9)
With nonhousehold members	374 (34.4)	213 (38.4)	161 (30.1)
Contact with acute respiratory illness cases	n = 1,087	n = 547	n = 540
None	971 (89.3)	449 (82.1)	522 (96.7)
At work only	66 (6.1)	54 (9.9)	12 (2.2)
At home only	44 (4.0)	38 (6.9)	6 (1.1)
At home and work	6 (0.6)	6 (1.1)	0
Attended gatherings	n = 1,113	n = 564	n = 549
Attended in preceding 2 weeks	113 (10.2)	50 (8.9)	63 (11.5)

*Clinic participants are those recruited on clinic premises, where they might have been seeking care for COVID-19 or any other illness. Outreach participants are those recruited at mobile testing operations in the community, who were not seeking medical care. SARS-CoV-2, severe acute respiratory syndrome coronavirus 2.

We estimated period-specific prevalence of SARS-CoV-2 infection and seropositivity, accounting for inverse sampling weights, by using a generalized linear model with a log-binomial link function. Models accounted for the 4 recruitment periods, presence of any symptoms, and recruitment venue. We used the model parameter estimates to summarize period-specific prevalence of TMA-positive and seropositive status for persons with and without symptoms whom we would expect to reach via community outreach. To account for missing data (1.1% of observations across all outcome and predictor variables), we sampled estimates from 5 independent iterations of the analysis carried out on multiple-imputed datasets. We conducted analyses in R version 4.0.3 (R Foundation for Statistical Computing, https://www.r-project.org); we used the Amelia II package (*21*) for multiple imputation and fit the multinomial logistic model using the nnet package (*22*).

## Results

### Clinical Surveillance Study

**Figure 1:**
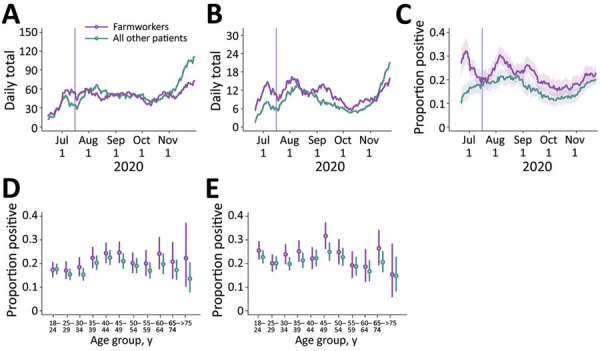
Cases of severe acute respiratory syndrome coronavirus 2 (SARS-CoV-2) diagnosed at Clínica de Salud del Valle de Salinas (CSVS), Monterey County, California, USA, June 15–November 30, 2020. We plotted the 2-week moving averages of the number of patients tested by CSVS (A); the number of SARS-CoV-2 infections diagnosed (B); and the proportion of tests yielding positive results (C). Shading indicates 95% CIs. Vertical lines indicate the date the cross-sectional study began, July 16. We also plotted age- and sex- stratified test-positive fractions for female (D) and male (E) patients. Bars indicate ranges; circles indicate medians.

During June 15–November 30, CSVS administered 14,169 SARS-CoV-2 TMA tests to adults, including 6,864 tests among farmworkers and 7,305 among other adults living in the same communities (Figure 1, panel A). In total, 1,514 (22.1%) tests among farmworkers had positive results, compared with 1,255 (17.2%) among other adults in the same communities, which corresponds to a 28.5% (95% CI 20.1%–37.4%) higher probability of positive test results among farmworkers (Figure 1, panels B, C). The test-positive fraction was similarly higher among men than among women, for both farmworkers (men 23.7% vs. women 20.5%; risk ratio [RR] 1.16, 95% CI 1.06–1.27) and nonfarmworkers (men 21.7% vs. women 18.8%; RR 1.15, 95% CI 1.09–1.23). Point estimates of the test-positive fraction were consistent with equal or higher prevalence of infection among farmworkers across all age and sex strata (Figure 1, panels D, E).

Among farmworkers, multiple peaks in the proportion of TMA tests yielding positive results were evident, with the moving average of the test-positive fraction reaching 32.0% (95% CI 27.2%–37.0%) over the period of June 23–July 7 and 30.4% (95% CI 27.0%–34.0%) over the period of August 7–21 (Figure 1, panel C). After declining from mid-September to early October, both the number of tests and the proportion yielding positive results increased through the remainder of the study period. During October 10–November 23, the 2-week moving average of the number of tests conducted daily increased from 35.5 to 69.5 among farmworkers and from 38.7 to 104.5 among other adults. The proportion positive tests increased from 15.4% (95% CI 12.2%–18.8%) to 22.7% (95% CI 20.0%–25.5%) among farmworkers and from 12.1% (95% CI 9.4%–15.1%) to 19.9% (95% CI 17.9%–22.1%) among other adults. This increase in case volume among nonfarmworker adults in November, without a commensurate rise among farmworkers, coincided with the annual migration of many Salinas Valley farmworkers to Yuma, Arizona, and elsewhere (*23*).

### Cross-Sectional Study

**Figure 2:**
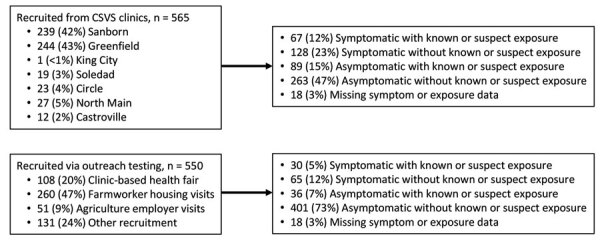
Participants recruited into the cross-sectional study of coronavirus disease (COVID-19) among farmworkers, Monterey County, California, USA, July 16–November 30, 2020. Number of farmworkers recruited at each participating CSVS clinic and outreach venues in the community. Number and proportion of participants reporting symptoms or exposure to known or suspected COVID-19 cases during the prior 2 weeks for both the clinic-based and outreach samples. CSVS, Clínica de Salud del Valle de Salinas.

Our cross-sectional study recruited 1,115 farmworkers, including 565 who were tested at clinics and 550 tested through outreach efforts (Figure 2). SARS-CoV-2 TMA test results were obtained for 1,111 (99.6%) participants and ELISAs conducted for 1,058 (94.9%) participants (Table 2). Most of the farmworkers in this study were born in Mexico, spoke Spanish at home, had primary school-level education or less, earned <25,000 $US per year (Table 2), and worked in the fields (Table 1); 36.3% lived in crowded housing (Table 1). Most (81.8%) were overweight or obese, but only 4.4% were current smokers (Table 1). Compared with farmworkers recruited via outreach, farmworkers recruited at clinics had lower levels of educational attainment and had been in the United States fewer years. More spoke indigenous languages at home (14.9% vs. 4.7%; Table 2) and reported contact with an individual experiencing respiratory symptoms in the 2 weeks prior to testing (17.9% vs. 3.3%; Table 1).

**Table 2:** Demographic characteristics, socioeconomic status, and SARS-CoV-2 infection among persons recruited for cross-sectional study of farmworkers, Monterey County, California, USA, July 16–November 30, 2020*

Characteristics	Enrollees, no. (%)		
	All, n = 1,115	Clinic, n = 565	Outreach, n = 550
Age range, y			
18–29	277 (24.8)	140 (24.7)	137 (24.9)
30–39	274 (24.6)	136 (24.0)	138 (25.1)
40–49	298 (26.7)	163 (28.8)	135 (24.5)
50–59	200 (17.9)	90 (15.9)	110 (20.0)
>60	66 (5.9)	36 (6.4)	30 (5.5)
Sex			
F	586 (52.6)	302 (53.5)	284 (51.6)
M	529 (47.4)	263 (46.5)	266 (48.4)
Country of birth			
Mexico	929 (83.3)	486 (86.0)	443 (80.5)
United States	142 (12.7)	49 (8.7)	93 (16.9)
Other	44 (3.9)	30 (5.3)	14 (2.5)
Language spoken at home			
Spanish	948 (85.0)	460 (81.4)	488 (88.7)
English	57 (5.1)	21 (3.7)	36 (6.5)
Indigenous language	110 (9.9)	84 (14.9)	26 (4.7)
Education	n = 1,114	n = 564	n = 550
Never attended school	62 (5.6)	48 (8.5)	14 (2.5)
Some primary school	430 (38.6)	229 (40.5)	201 (36.5)
Primary school complete	238 (21.3)	119 (21.1)	119 (21.6)
Some high school	142 (12.7)	68 (12.0)	74 (13.5)
High school complete	242 (21.7)	100 (17.7)	142 (25.8)
Family income, US $	n = 1,059	n = 536	n =523
<25,000	560 (52.8)	291 (54.3)	269 (51.4)
25,000–34,999	260 (24.6)	112 (20.9)	148 (28.3)
35,000–49,999	162 (15.3)	86 (16.0)	76 (14.5)
>50,000	77 (7.3)	47 (8.8)	30 (5.7)
Years in United States	n = 1,114	n = 564	n = 550
<15	262 (26.9)	157 (30.4)	105 (23.0)
15–19	194 (19.9)	110 (21.3)	84 (18.4)
20–29	299 (30.7)	141 (27.3)	158 (34.6)
>30	217 (22.3)	107 (20.7)	110 (24.1)
H2A visa holder	n = 960	n = 509	n = 451
Holds H2A visa	65 (6.8)	20 (4.0)	45 (10.0)
Body mass index	n = 1,087	n = 545	n = 542
<18.5, underweight	4 (0.4)	2 (0.4)	2 (0.4)
18.5–24.9, normal	194 (17.8)	106 (19.4)	88 (16.2)
25–29.9 overweight	423 (38.9)	212 (38.9)	211 (38.9)
>30, obese	466 (42.9)	225 (41.3)	241 (44.5)
Smoking	n = 1,114	n = 564	n = 550
Never smoked	907 (81.4)	460 (81.6)	447 (81.3)
Former smoker	158 (14.2)	86 (15.2)	72 (13.1)
Current smoker	49 (4.4)	18 (3.2)	31 (5.6)
Recent COVID-19 symptoms	n = 1,108	n = 565	n = 543
Symptoms in preceding 2 weeks	301 (27.2)	200 (35.8)	101 (18.4)
History of COVID-19 symptoms	n = 1,108	n = 558	n = 550
Symptoms since pandemic started in December 2019	457 (41.2)	266 (47.7)	191 (34.7)
SARS-CoV-2 infection	n = 1,111	n = 563	n = 548
Positive TMA result	141 (12.7)	105 (18.7)	36 (6.6)
Prior SARS-CoV-2 infection	n = 1,058	n = 526	n = 532
Positive antibody result	201 (19.0)	97 (18.4)	104 (19.5)

*Clinic participants are those recruited on clinic premises, where they might have been seeking care for COVID-19 or any other illness. Outreach participants are those recruited at mobile testing operations in the community, who were not seeking medical care. COVID-19, coronavirus disease; SARS-CoV-2, severe acute respiratory syndrome coronavirus 2; TMA, transcription-mediated amplification nucleic acid assay.

Overall, 27.2% of participants reported symptoms potentially related to COVID-19 in the previous 2 weeks and 41.2% reported symptoms since the start of the pandemic (Table 3). A higher proportion of farmworkers recruited at clinics compared with those recruited via outreach reported >1 symptom potentially attributable to COVID-19 in either the 2 weeks before testing (35.8% vs. 18.4%) or the period since December 2019 (47.7% vs. 34.7%) (Table 2). Among all farmworkers, 12.7% tested TMA-positive for current SARS-CoV-2 infection, including 18.7% of farmworkers tested at clinics and 6.6% of those tested via outreach (Table 2). In contrast, 19.0% of farmworkers tested via ELISA were found to have antibody evidence of prior infection; similar prevalence was found among those tested in the clinics (18.4%) and via outreach (19.4%).

**Table 3:** Prevalence of COVID-19 symptoms and severe acute respiratory syndrome coronavirus 2 infection among farm workers enrolled in a cross-sectional study, Monterey County, California, USA, July 16–November 30, 2020*

Symptoms	All participants, n = 1,108			Clinic participants, n = 558			Outreach participants, n = 550	
	Frequency	Infected		Frequency	Infected		Frequency	Infected
Any symptom	301 (27.2)	83 (27.7)		200 (35.8)	68 (34.2)		101 (18.4)	15 (14.9)
No symptoms	807 (72.8)	57 (7.1)		358 (64.2)	36 (10.1)		449 (81.6)	21 (4.7)
Nonproductive cough	68 (6.1)	31 (45.6)		47 (8.4)	26 (55.3)		21 (3.8)	5 (23.8)
Productive cough	68 (6.1)	25 (37.3)		51 (9.1)	21 (42.0)		17 (3.1)	4 (23.5)
Pain or pressure in the ears	24 (2.2)	10 (41.7)		19 (3.4)	10 (52.6)		5 (0.9)	0
Blocked nose	62 (5.6)	22 (36.1)		50 (9.0)	19 (38.8)		12 (2.2)	3 (25.0)
Runny nose	78 (7.0)	24 (31.2)		56 (10.0)	18 (32.7)		22 (4.0)	6 (27.3)
Sneezing	95 (8.6)	21 (22.3)		61 (10.9)	16 (26.7)		34 (6.2)	5 (14.7)
Watery eyes	57 (5.1)	14 (25.0)		48 (8.6)	14 (29.8)		9 (1.6)	0
Hoarseness	49 (4.4)	19 (38.8)		42 (7.5)	17 (40.5)		7 (1.3)	2 (28.6)
Self-reported fever†	56 (5.1)	33 (58.9)		47 (8.4)	29 (61.7)		9 (1.6)	4 (44.4)
Sweating	48 (4.3)	22 (45.8)		40 (7.2)	20 (50.0)		8 (1.5)	2 (25.0)
Chills	74 (6.7)	35 (47.3)		63 (11.3)	33 (52.4)		11 (2.0)	2 (18.2)
Headache	147 (13.3)	46 (31.5)		100 (17.9)	39 (39.4)		47 (8.5)	7 (14.9)
Tickle in throat	49 (4.4)	17 (34.7)		36 (6.5)	15 (41.7)		13 (2.4)	2 (15.4)
Sore throat	103 (9.3)	32 (31.1)		78 (14.0)	29 (37.2)		25 (4.5)	3 (12.0)
Myalgia	97 (8.8)	44 (45.8)		79 (14.2)	40 (51.3)		18 (3.3)	4 (22.2)
Chest pain	26 (2.3)	11 (42.3)		21 (3.8)	10 (47.6)		5 (0.9)	1 (20.0)
Sinus pain	17 (1.5)	7 (41.2)		14 (2.5)	7 (50.0)		3 (0.5)	0 (0.0)
Swollen glands	18 (1.6)	5 (27.8)		11 (2.0)	5 (45.5)		7 (1.3)	0 (0.0)
Loss of appetite	38 (3.4)	21 (55.3)		32 (5.7)	18 (56.2)		6 (1.1)	3 (50.0)
Difficulty breathing	34 (3.1)	18 (52.9)		27 (4.8)	16 (59.3)		7 (1.3)	2 (28.6)
Wheezing	15 (1.4)	6 (40.0)		12 (2.2)	6 (50.0)		3 (0.5)	0
Shortness of breath	22 (2.0)	18 (81.8)		19 (3.4)	16 (84.2)		3 (0.5)	2 (66.7)
Diarrhea	40 (3.6)	15 (37.5)		33 (5.9)	14 (42.4)		7 (1.3)	1 (14.3)
Nausea	39 (3.5)	13 (33.3)		32 (5.7)	13 (40.6)		7 (1.3)	0
Stomach pain	47 (4.2)	15 (31.9)		34 (6.1)	12 (35.3)		13 (2.4)	3 (23.1)
Trouble thinking	18 (1.6)	5 (27.8)		10 (1.8)	5 (50.0)		8 (1.5)	0 (0.0)
Fatigue	94 (8.5)	33 (35.5)		70 (12.5)	31 (44.9)		24 (4.4)	2 (8.3)
Loss of sense of taste	33 (3.0)	22 (66.7)		26 (4.7)	18 (69.2)		7 (1.3)	4 (57.1)
Loss of sense of smell	32 (2.9)	22 (68.8)		25 (4.5)	19 (76.0)		7 (1.3)	3 (42.9)
Pain or pressure in the eyes	25 (2.3)	6 (24.0)		16 (2.9)	6 (37.5)		9 (1.6)	0

*Testing was performed by using transcription-mediated amplification (TMA) assay. Percentages were calculated excluding persons for whom data were not available. Clinic participants are those recruited on clinic premises, where they might have been seeking care for COVID-19 or any other illnesses. Outreach participants are those recruited at mobile testing operations in the community, who were not seeking medical care. For frequency, proportions are computed among all tested. For infected, proportions indicate the prevalence of current, TMA-positive infection among those with the indicated symptom(s) in the previous 2 weeks. COVID-19, coronavirus disease. †Participants were not asked to verify whether they recorded their body temperature.

**Figure 3:**
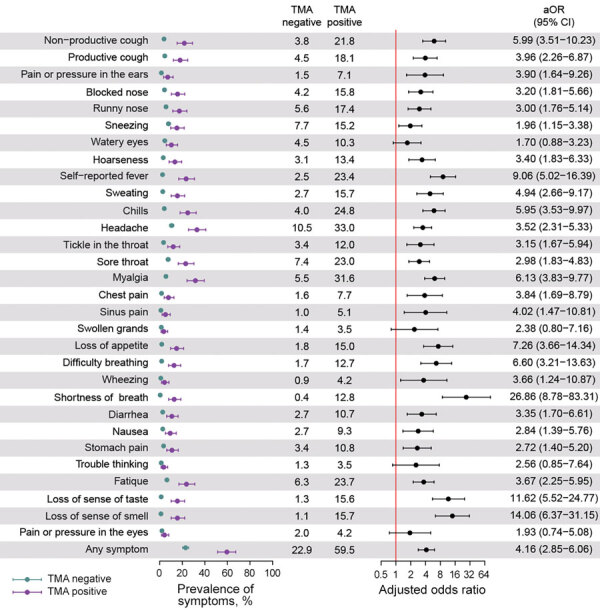
Association of symptoms and current TMA-positive severe acute respiratory syndrome coronavirus 2 (SARS-CoV-2) infection in cross-sectional study of farmworkers, Monterey County, California, USA, July 16–November 30, 2020. Illustration of the prevalence of each symptom during the 2 weeks preceding testing among persons who tested positive and negative for SARS-CoV-2 infection via TMA and the aOR conveying the association of each symptom with current infection. We used logistic regression to determine aORs, controlling for age group, sex, and recruitment venue (i.e., clinic-based or outreach sample). Bars denote 95% CIs around point estimates (circles). aOR, adjusted odds ratio; TMA, transcription-mediated amplification nucleic acid assay.

Of all farmworkers who had TMA-positive test results, 58.9% reported symptoms in the preceding 2 weeks, including 64.8% among those recruited from the clinic and 41.7% of those recruited via outreach (Table 3). Overall, 27.2% of those who had any potential COVID-19 symptoms in the 2 weeks before enrollment had current TMA-positive SARS-CoV-2 infection. Prevalence of current infection among farmworkers recruited in the clinic was 34.2% for those reporting any symptoms and prevalence was 10.1% for those reporting no symptoms. Among farmworkers recruited from outreach testing, current TMA-positive SARS-CoV-2 infection was detected in 14.9% of those reporting any symptoms and 4.7% among those reporting no symptoms (Table 3). After adjustment for age, sex, and recruitment setting, the aOR of a TMA-positive SARS-CoV-2 test result was 4.16 (95% CI 2.85–6.06) among farmworkers reporting any of the solicited symptoms in the previous 2 weeks compared with those reporting no symptoms (Figure 3). 

Symptoms most strongly associated with current SARS-CoV-2 infection included shortness of breath (aOR 26.86, 95% CI 8.78–83.31), loss of smell (aOR 14.06, 95% CI 6.37–31.15), loss of taste (aOR 11.62, 95% CI 5.52–24.77), and self-reported fever (aOR 9.06, 95% CI 5.02–16.39). Each of these symptoms, however, was reported by <25% of persons with current SARS-CoV-2 infection. For the most commonly reported symptoms among persons testing positive, headache (33.0%) was associated with 3.52-fold (95% CI 2.31–5.33) higher adjusted odds of SARS-CoV-2 RNA detection, and myalgia (31.6%) was associated with 6.13-fold (95% CI 3.83–9.77) higher adjusted odds.

**Figure 4:**
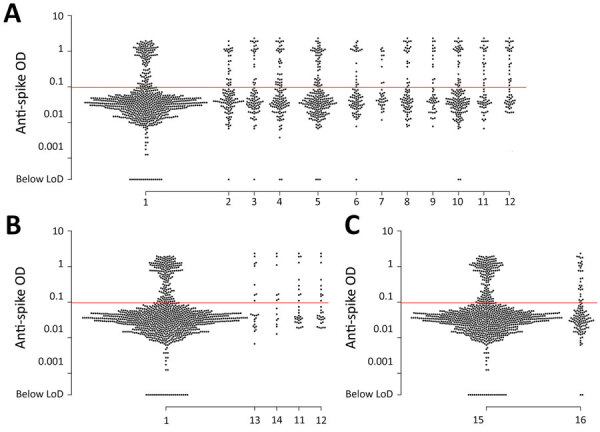
Scatter plot of anti-spike IgG reactivity and association with recalled coronavirus disease (COVID-19) symptoms in a cross-sectional study of farmworkers, Monterey County, California, USA, July 16–November 30, 2020. A) Reactivity among persons who reported experiencing or not experiencing various symptoms potentially associated with COVID-19 since December 2019: 1, none of the symptoms listed here; 2, blocked nose (p = 0.027); 3, sweating (p = 0.010); 4, chills (p = 0.013); 5, headache (p = 0.034); 6, tickling in throat (p = 0.029); 7, sinus pain or pressure (p = 0.034); 8, loss of appetite (p<0.001); 9, shortness of breath (p = 0.006); 10, fatigue (p = 0.032); 11, loss of taste (p<0.001); 12, loss of smell (p<0.001). B) Reactivity among persons who reported experiencing or not experiencing various symptoms in the 2 weeks before enrollment (data not shown for symptoms with p>0.1): 1, none of the symptoms listed here; 13, chest pain (p = 0.061); 14, wheezing (p = 0.043); 11, loss of taste (p = 0.037); 12, loss of smell (p = 0.072). C) Reactivity among persons who had a positive or negative severe acute respiratory syndrome coronavirus 2 transcription-mediated amplification (TMA) nucleic acid assay result at the enrollment visit: 15, TMA-positive (p = 0.325); 16 TMA-negative. Reported p values are measured in logistic regression models with the occurrence of each symptom as the outcome and antibody ELISA OD values (log-transformed) as predictors and adjusted for age group and sex. Red lines indicate assay LoD. LoD, limit of detection; OD, optical density.

Persons who recalled experiencing a blocked nose, sweating, chills, headache, a tickling sensation in the throat, a feeling of pain or pressure in the sinuses, loss of appetite, shortness of breath, fatigue, loss of taste, or loss of smell since December 2019 had higher antibody reactivity, on average, than persons who did not recall experiencing such symptoms (Figure 4, panel A). We also identified higher antibody reactivity among persons experiencing wheezing or loss of taste in the preceding 2 weeks, and suggestive associations of higher antibody measurements with persons reporting chest pain and loss of smell in the preceding 2 weeks (Figure 4, panel B). We found no statistically significant difference in quantitative antibody reactivity measures among persons who were currently infected with SARS-CoV-2 compared with persons who were not (p = 0.3), suggesting associations of antibody reactivity with recent symptoms were not attributable to current infection. Among 129 TMA-positive cases 30 (18%) met the threshold for IgG seropositivity, as did 168/925 (23%) TMA-negative cases.

**Figure 5:**
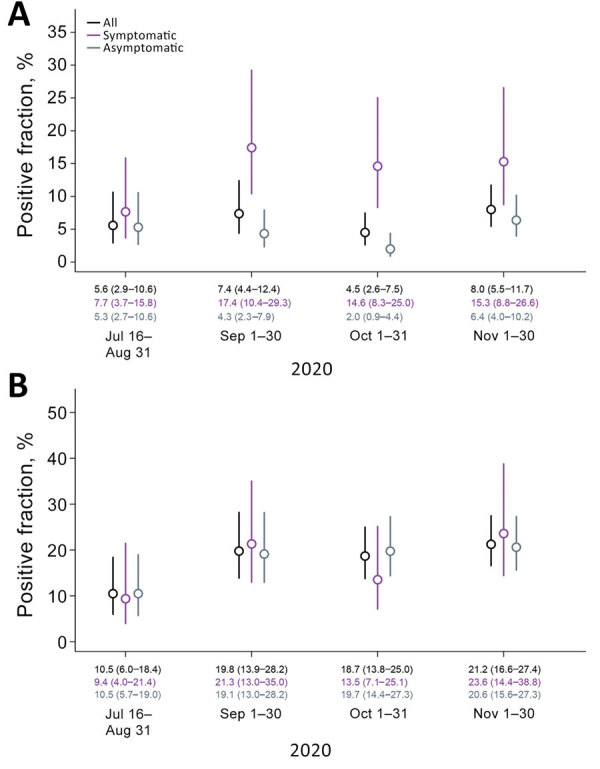
Prevalence of severe acute respiratory syndrome coronavirus 2 (SARS-CoV-2) positivity by transcription-mediated amplification (TMA) and seropositivity over time, Monterey County, California, USA, July 16–November 30, 2020. A) SARS-CoV-2 TMA; B) SARS-CoV-2 IgG ELISA. Estimated prevalence of SARS-CoV-2 infection and seropositivity in a sample population reached by outreach testing, reweighted to correct for differences in the population seeking testing over the course of the study. Lines delineate 95% CI around mean estimates (circles); medians and 95% CIs appear along the baseline.

Reweighting the sample to adjust for differences among persons tested over time, we estimated the prevalence of current, TMA-positive SARS-CoV-2 infection within the population reached by outreach testing was 5.6% (95% CI 2.9%–10.6%) during July 16–August 31, 7.4% (95% CI 4.4%–12.4%) during September 1–30, 4.5% (95% CI 2.6%–7.5%) during October 1–31, and 8.0% (95% CI 5.5%–11.7%) during November 1–30 (Figure 5, panel A). These results closely tracked patterns in the proportion of tests yielding positive results among all farmworkers tested by CSVS (Figure 1, panel C). Over this period, we estimated 2.0% (95% CI 0.9%–4.4%) to 6.4% (95% CI 4.0%–10.2%) prevalence of current SARS-CoV-2 infection among asymptomatic persons and 7.7% (95% CI 3.7%–15.8%) to 17.4% (95% CI 10.4%–29.3%) prevalence of current SARS-CoV-2 infection among persons experiencing >1 symptom. Estimated seroprevalence increased from 10.5% (95% CI 6.0%–18.4%) to 21.2% (95% CI 16.6%–27.4%) over the duration of the study, with similar results among symptomatic and asymptomatic persons during each period (Figure 5, panel B).

## Discussion

Among all adults tested for SARS-CoV-2 infection by clinics serving the Monterey County farmworker population, test positivity was 28% higher for farmworkers than for nonfarmworkers from the same communities. Test positivity was much higher (22.1%) among farmworkers tested by CSVS compared with the overall test-positive fraction (6.1%) observed in Monterey County over the same period (*24*). Within the cross-sectional study subpopulation, we identified sustained high prevalence of infection: TMA-positive results among 6.6% of persons tested in the community and 18.7% of those tested in clinics. We estimated ≈10% of the farmworker population became infected over a 3-month period during the study, yielding ≈21% seroprevalence by November 2020. This seroprevalence is well above the 5% seroprevalence noted among California adults in a large-scale assessment of blood specimens submitted for routine clinical screening or clinical management in September (*25*). A previous study in San Francisco likewise identified elevated infection risk in an urban low-income and predominantly Latino population, with 6.0% prevalence of current infection among frontline workers and 7.7% seroprevalence by late April 2020 (*26*). Our findings demonstrate high infection risk among farmworkers during the ongoing pandemic.

We identified a diverse array of symptoms, including gastrointestinal and other nonrespiratory symptoms, associated with SARS-CoV-2 infection. Among persons found to be TMA-positive for current SARS-CoV-2 infection in our study, 41% did not report experiencing any symptoms in the 2 weeks preceding their test. Similar results have been reported in other studies (*27*). Of note, persons could have been presymptomatic at the time of their interview; in addition, asymptomatic persons who seek testing might not represent the broader community (for instance, if testing is triggered by known SARS-CoV-2 exposure). The ≈2%–6% prevalence of infection among persons without symptoms in the community suggests substantial risk for exposure to clinically inapparent cases. Guidance issued for growers to screen farmworkers for fever or other COVID-19 symptoms likely is inadequate to prevent workplace infections (*28*). We also identified associations of higher antibody reactivity with current symptoms, including loss of taste and smell, chest pain, and wheezing. Participants in our study likely experienced these symptoms in a persisting manner beyond the acute infectious stage because seroconversion typically occurs 8–14 days after initial symptoms (*29*). The clinical profile of long COVID has not been fully clarified, but the same symptoms we noted have been identified as prominent complaints in prior studies of prolonged COVID-19 illness, along with fatigue, joint pain, and headache (*30*,*31*; C.H. Sudra et al., unpub. data, https://doi.org/10.1101/2020.10.19.20214494).

Our study’s limitations include that we cannot verify how well our sample represents the farmworker population, many of whom are hidden from population statistical measures (*32*); our findings should be taken to represent persons reached by testing. Because we excluded persons who did not speak Spanish or English well enough to participate in the cross-sectional study, our study likely underrepresents indigenous populations, which are estimated to be 13% of Salinas Valley farmworkers (*11*). Roughly half of our cross-sectional study participants were enrolled in clinic-based testing, among whom infection prevalence was higher. For this reason, our statistical framework accounted for differences between clinic-based and outreach samples. Last, waning antibody titers from infections acquired early in the pandemic might have contributed to underestimation of seroprevalence, particularly among persons who experienced mild or asymptomatic infection (*33*).

The recommendation of the Advisory Committee on Immunization Practices prioritized residents of long-term care facilities and healthcare workers for phase 1 vaccination programs (*34*), but prioritization of differing essential workforce groups among phase 2 recipients will be determined by states. Our study demonstrates high risk for SARS-CoV-2 infection, and both acute and persisting COVID-19 symptoms, among farmworkers in the Salinas Valley. These findings underscore the need to deliver vaccination and other preventive interventions to help reduce further illness among farmworkers and mitigate spread of COVID-19 in the United States.

This article was preprinted at https://doi.org/10.1101/2020.12.27.20248894. 

## References

[1] Lewnard JA, Lo NC. Scientific and ethical basis for social-distancing interventions against COVID-19. Lancet Infect Dis. 2020;20:631–3. 10.1016/S1473-3099(20)30190-0 32213329 PMC7118670

[2] The President’s Coronavirus Guidelines for America. 30 days to slow the spread. 2020 Mar 16 [cited 2021 Jan 21]. https://trumpwhitehouse.archives.gov/wp-content/uploads/2020/03/03.16.20_coronavirus-guidance_8.5x11_315PM.pdf

[3] Dyal JW, Grant MP, Broadwater K, Bjork A, Waltenburg MA, Gibbins JD, et al. COVID-19 among workers in meat and poultry processing facilities—19 states, April 2020. MMWR Morb Mortal Wkly Rep. 2020;69:69. 10.15585/mmwr.mm6918e3 32379731

[4] Steinberg J, Kennedy ED, Basler C, Grant MP, Jacobs JR, Ortbahn D, et al. COVID-19 outbreak among employees at a meat processing facility—South Dakota, March–April 2020. MMWR Morb Mortal Wkly Rep. 2020;69:1015–9. 10.15585/mmwr.mm6931a2 32759914 PMC7454899

[5] Stephenson J. COVID-19 Outbreaks among food production workers may intensify pandemic’s disproportionate effects on people of color. JAMA Heal Forum. 2020 Jun 19 [Epub ahead of print]. 10.1001/jamahealthforum.2020.0783 36218521

[6] Martin P. Immigration and farm labor: from unauthorized to H-2A for some? Migration Policy Institute. 2017 [cited 2021 Jan 21]. https://www.migrationpolicy.org/research/immigration-and-farm-labor-unauthorized-h-2a-some

[7] Barham BL, Melo A, Hertz T. Earnings, wages, and poverty outcomes of US farm and low-skill workers. Appl Econ Perspect Policy. 2020;42:307–34. 10.1002/aepp.13014

[8] Kerwin D, Warren RUS. Foreign-born workers in the global pandemic: essential and marginalized. J Migr Hum Secur. 2020;8:282–300. 10.1177/2331502420952752

[9] Becot F, Inwood S, Bendixsen C, Henning-Smith C. Health care and health insurance access for farm families in the United States during COVID-19: essential workers without essential resources? J Agromed. 2020;25:374–7. 10.1080/1059924X.2020.1814924 32921286 PMC11075044

[10] Carlisle-Cummins I. COVID-19 farmworker study (COFS): Historic pandemic worsens vulnerability of essential workers who feed us all. 2020 [cited 2021 Jan 21]. http://covid19farmworkerstudy.org/preliminary-data

[11] Villarejo D, Wadsworth G. Farmworker housing study and action plan for Salinas Valley and Pajaro Valley, April 2018. California Institute for Rural Studies. 2018 [cited 2021 Jan 21]. https://www.co.monterey.ca.us/home/showdocument?id=63729

[12] Eskenazi B, Bradman A, Gladstone EA, Jaramillo S, Birch K, Holland N. CHAMACOS, a longitudinal birth cohort study: lessons from the fields. J Child Health. 2003;1:3–27. 10.3109/713610244

[13] Bradman A, Chevrier J, Tager I, Lipsett M, Sedgwick J, Macher J, et al. Association of housing disrepair indicators with cockroach and rodent infestations in a cohort of pregnant Latina women and their children. Environ Health Perspect. 2005;113:1795–801. 10.1289/ehp.7588 16330367 PMC1314924

[14] Goldman L, Eskenazi B, Bradman A, Jewell NP. Risk behaviors for pesticide exposure among pregnant women living in farmworker households in Salinas, California. Am J Ind Med. 2004;45:491–9. 10.1002/ajim.20012 15164393

[15] Villarejo D. California’s hired farm workers move to the cities: the outsourcing of responsibility for farm labor housing. Presented at California Rural Legal Assistance Priorities Conference; Asilomar, California; July 16, 2013 [cited 2021 Jan 21]. https://www.crla.org/sites/all/files/u6/2014/rju0214/VillarejoFrmLbrHsngHlth_CRLA_012414.pdf

[16] Flocks J. The potential impact of COVID-19 on H-2A agricultural workers. J Agromed. 2020;25:367–9. 10.1080/1059924X.2020.1814922 32856557

[17] Smith E, Zhen W, Manji R, Schron D, Duong S, Berry GJ. Analytical and clinical comparison of three nucleic acid amplification tests for SARS-CoV-2 detection. J Clin Microbiol. 2020;58:e01134–20. 10.1128/JCM.01134-20 32571894 PMC7448672

[18] Skittrall JP, Wilson M, Smielewska AA, Parmar S, Fortune MD, Sparkes D, et al. Specificity and positive predictive value of SARS-CoV-2 nucleic acid amplification testing in a low-prevalence setting. Clin Microbiol Infect. 2020;S1198-743X(20)30614-5; [Epub ahead of print]. 33068757 10.1016/j.cmi.2020.10.003 PMC7554481

[19] Amanat F, Stadlbauer D, Strohmeier S, Nguyen THO, Chromikova V, McMahon M, et al. A serological assay to detect SARS-CoV-2 seroconversion in humans. Nat Med. 2020;26:1033–6. 10.1038/s41591-020-0913-5 32398876 PMC8183627

[20] Cole SR, Hernán MA. Constructing inverse probability weights for marginal structural models. Am J Epidemiol. 2008;168:656–64. 10.1093/aje/kwn164 18682488 PMC2732954

[21] Honaker J, King G, Blackwell M. Amelia II: a program for missing data. J Stat Softw. 2011;45:1–47. 10.18637/jss.v045.i07

[22] Ripley B, Venables W, Ripley MP. Package ‘nnet.’ 2016 [cited 2021 Jan 21]. https://cran.r-project.org/web/packages/nnet/nnet.pdf

[23] Matthews J. Salinas and Yuma share a common (salad) bowl. San Francisco Chronicle. 2018 Oct 20 [cited 2021 Jan 21]. https://www.sfchronicle.com/opinion/article/Salinas-and-Yuma-share-a-common-salad-bowl-13319515.php

[24] Moreno E. COVID-19 data, metrics and updates. Monterey County Health Department, Public Health Bureau. 2020 [cited 2021 Jan 21]. https://www.co.monterey.ca.us/home/showdocument?id=96259

[25] Havers FP, Reed C, Lim T, Montgomery JM, Klena JD, Hall AJ, et al. Seroprevalence of antibodies to SARS-CoV-2 in 10 sites in the United States, March 23–May 12, 2020. JAMA Intern Med. 2020 Jul 21 [Epub ahead of print]. 10.1001/jamainternmed.2020.4130 PMC12507447 32692365

[26] Chamie G, Marquez C, Crawford E, Peng J, Petersen M, Schwab D, et al.; CLIAhub Consortium. SARS-CoV-2 Community Transmission disproportionately affects Latinx population during Shelter-in-Place in San Francisco. Clin Infect Dis. 2020;ciaa1234; Epub ahead of print. 10.1093/cid/ciaa1234 32821935 PMC7499499

[27] Buitrago-Garcia D, Egli-Gany D, Counotte MJ, Hossmann S, Imeri H, Ipekci AM, et al. Occurrence and transmission potential of asymptomatic and presymptomatic SARS-CoV-2 infections: A living systematic review and meta-analysis. PLoS Med. 2020;17:e1003346. 10.1371/journal.pmed.1003346 32960881 PMC7508369

[28] Advisory for agricultural worker protection during COVID-19 crisis on the Central Coast of California. Monterey County Health Department 2020 [cited 2021 Jan 21]. https://www.co.monterey.ca.us/home/showdocument?id=88063

[29] Long QX, Liu BZ, Deng HJ, Wu GC, Deng K, Chen YK, et al. Antibody responses to SARS-CoV-2 in patients with COVID-19. Nat Med. 2020;26:845–8. 10.1038/s41591-020-0897-1 32350462

[30] Mandal S, Barnett J, Brill SE, Brown JS, Denneny EK, Hare SS, et al.; ARC Study Group. ‘Long-COVID’: a cross-sectional study of persisting symptoms, biomarker and imaging abnormalities following hospitalisation for COVID-19. Thorax. 2020;thoraxjnl-2020-215818; Epub ahead of print. 10.1136/thoraxjnl-2020-215818 33172844 PMC7615158

[31] Carfì A, Bernabei R, Landi F; Gemelli Against COVID-19 Post-Acute Care Study Group. Persistent symptoms in patients after acute COVID-19. JAMA. 2020;324:603–5. 10.1001/jama.2020.12603 32644129 PMC7349096

[32] Bail KM, Foster J, Dalmida SG, Kelly U, Howett M, Ferranti EP, et al. The impact of invisibility on the health of migrant farmworkers in the southeastern United States: a case study from georgia. Nurs Res Pract. 2012;2012:760418. 10.1155/2012/760418 22830007 PMC3395173

[33] Choe PG, Kang CK, Suh HJ, Jung J, Song KH, Bang JH, et al. Waning antibody responses in asymptomatic and symptomatic SARS-CoV-2 infection. Emerg Infect Dis. 2021;27:327–9. 10.3201/eid2701.203515 33050983 PMC7774548

[34] Dooling K; ACIP COVID-19 Vaccines Work Group. Phased allocation of COVID-19 vaccines. Presented at the ACIP Meeting 2020 Dec 1 [cited 2021 Jan 21]. https://www.cdc.gov/vaccines/acip/meetings/downloads/slides-2020-12/COVID-02-Dooling.pdf

